# Identification of cytotoxic markers in methamphetamine treated rat C6 astroglia-like cells

**DOI:** 10.1038/s41598-019-45845-1

**Published:** 2019-06-28

**Authors:** Ramesh B. Badisa, Chantel Wiley, Kesa Randell, Selina F. Darling-Reed, Lekan M. Latinwo, Maryam Agharahimi, Karam F. A. Soliman, Carl B. Goodman

**Affiliations:** 1 College of Pharmacy and Pharmaceutical Sciences, Florida A&M University, Tallahassee, FL 32307 USA; 2 Department of Biological Science, Florida A&M University, Tallahassee, FL 32307 USA

**Keywords:** Cellular neuroscience, Cell death

## Abstract

Methamphetamine (METH) is a powerfully addictive psychostimulant that has a pronounced effect on the central nervous system (CNS). The present study aimed to assess METH toxicity in differentiated C6 astroglia-like cells through biochemical and toxicity markers with acute (1 h) and chronic (48 h) treatments. In the absence of external stimulants, cellular differentiation of neuronal morphology was achieved through reduced serum (2.5%) in the medium. The cells displayed branched neurite-like processes with extensive intercellular connections. Results indicated that acute METH treatment neither altered the cell morphology nor killed the cells, which echoed with lack of consequence on reactive oxygen species (ROS), nitric oxide (NO) or inhibition of any cell cycle phases except induction of cytoplasmic vacuoles. On the other hand, chronic treatment at 1 mM or above destroyed the neurite-like processors and decreased the cell viability that paralleled with increased levels of ROS, lipid peroxidation and lactate, depletion in glutathione (GSH) level and inhibition at G0/G1 phase of cell cycle, leading to apoptosis. Pre-treatment of cells with N-acetyl cysteine (NAC, 2.5 mM for 1 h) followed by METH co-treatment for 48 h rescued the cells completely from toxicity by decreasing ROS through increased GSH. Our results provide evidence that increased ROS and GSH depletion underlie the cytotoxic effects of METH in the cells. Since loss in neurite connections and intracellular changes can lead to psychiatric illnesses in drug users, the evidence that we show in our study suggests that these are also contributing factors for psychiatric-illnesses in METH addicts.

## Introduction

METH, an amphetamine derivative, is one of the popular synthetic illegal psycho-stimulants abused not only in the US^1^ but also worldwide^2,3^. At present, there are about 10 million METH users in the US, and about 35 million worldwide^4–6^. Unfortunately, its use is gaining popularity again in the US^7,8^.

Crystal meth, ice and speed are some popular club names for METH, and it is portrayed as a poor man’s cocaine and the drug of choice for economically poor addicts. Though both METH and cocaine work as CNS stimulants, METH triggers much stronger pharmacological effect^9,10^ than cocaine because the latter is metabolized most rapidly and disposed of the body in contrast to the former^9^. Due to a different mechanism of action^3,9^, METH releases more dopamine in the brain as opposed to cocaine. For these reasons, METH is considered dangerously addictive.

While increased alertness, decreased appetite, hyperthermia, cerebrovascular accidents, and diarrhea^11^ are some of the acute effects of METH, weight loss, depression, agitation & insomnia^12^, neurotoxicity^13^ and cognitive sequelae^14–17^ are a few chronic psychiatric symptoms. The acute and chronic complications may be related to the toxic effects induced by METH at the cellular level in the CNS. In order to understand the mechanism of toxicity of METH, *in vitro* studies were conducted using various neuronal cell types due to METH interaction with neurons in the CNS^18–25^. However, not many studies have addressed the METH-induced toxic effect in astrocytes. Since astrocytes are considerably more abundant than neurons in many regions of mammalian brain^26,27^, it is possible that events of METH toxicity could manifest in these cells long before they die. It is not yet known what toxic markers METH induces in astrocytes. Therefore, identifying various toxic markers in astrocytes is imperative so that safe therapeutic strategies can be developed against the neurodegeneration associated with chronic use of METH.

The primary aim of our study is to discern the cytotoxic markers for METH using rat C6 astroglia-like cells. We tested these cells at acute (1 h) and chronic (48 h) time points. These cells behave like astrocytes in terms of expression of GFAP^28^, a marker protein in differentiated matured astrocytes^29,30^, and exhibit similarities to humans in terms of gene expression^31^ and enzymes^32^. The cytotoxic markers we focused on include vacuolation, viability, ROS, NO release, morphology, lipid peroxidation, lactate release, GSH level and apoptosis at acute and chronic treatments. Furthermore, the inhibitory role of METH on cell cycle phases was also assessed.

## Results

### Lack of acute METH effect on cells

**Figure 1:**
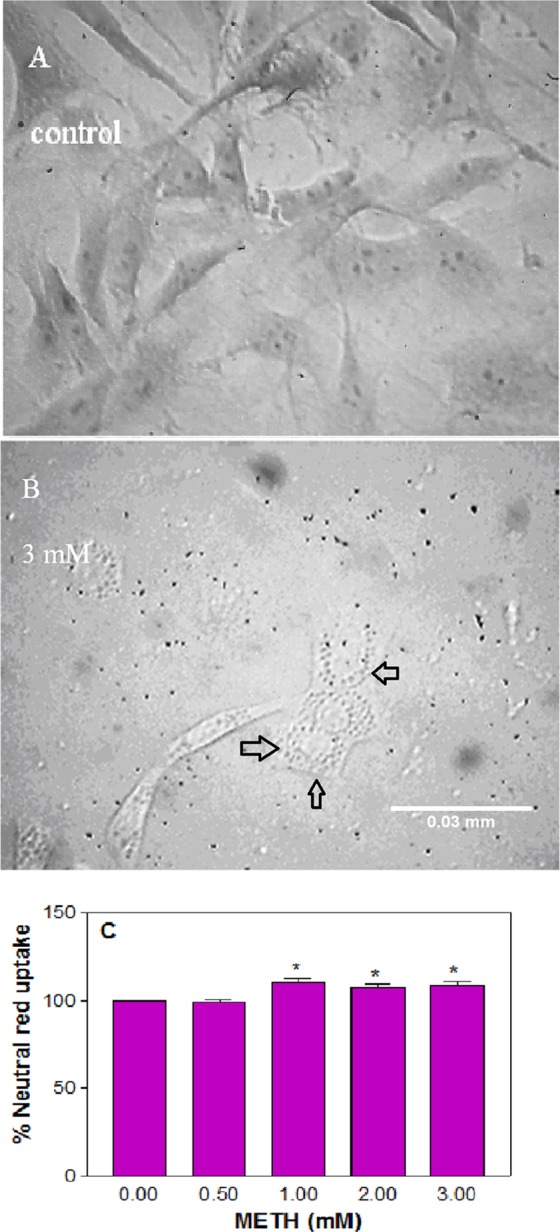
METH-induced vacuolation. C6 astroglia-like cells were treated with equal volume of vehicle (PBS control) or 0.5 to 3 mM METH for 1 h. Images of unstained cells were taken using an inverted phase contrast IX-70 Olympus microscope with a 40x objective. No vacuoles were seen in the control cells (**A**) but seen in 3 mM treated cells (**B**). Scale bar: 0.03 mm. For the sake of quantification of vacuoles (**C**), the cells were stained with neutral red for 10 min and quantified in a plate reader. Data were represented as mean ± SEM (*n* = 12), *P < 0.05, significant compared to control, one-way ANOVA, Dunnett’s multiple comparison test.

**Figure 2:**
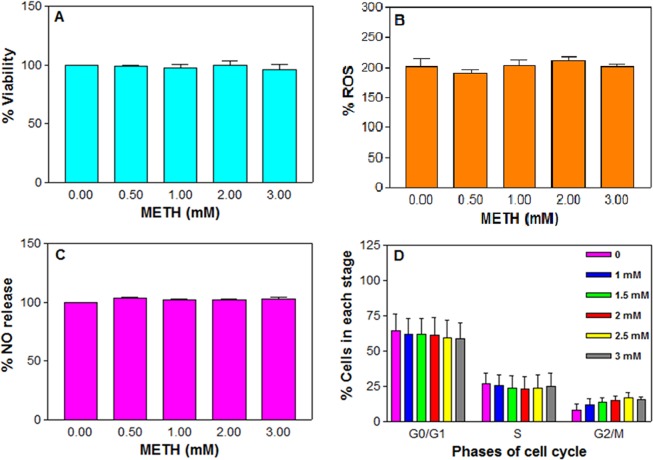
Effect of acute METH in C6 astroglia-like cells on viability (**A**), ROS generation (**B**), nitric oxide release (**C**) and cell cycle phases (**D**). Cells were treated with an equal volume of vehicle (PBS control) or 0.5 to 3 mM METH for 1 h. Cell viability was evaluated by a Celltiter 96 Aqueous one solution kit (*n* = 16). ROS generation was measured by staining the cells with an H_2_DCFDA dye (20 μM, 30 min), followed by measurement of DCF in a micro plate fluorometer with excitation and emission filters set at 485 and 530 nm respectively (*n* = 8–12). Nitric oxide release was assayed by Griess reagent (*n* = 12), and the cell cycle phases were analyzed by flow cytometry (*n* = 2). Data were represented as mean ± SEM, P > 0.05, insignificant compared to corresponding controls, one-way ANOVA, Dunnett’s multiple comparison test.

Acute treatment for 1 h was chosen based on an earlier report^28^. Initial treatment of the cells for 1 h at METH concentrations lower than 500 μM did not result in any cell death (data not shown). As reported on various cell types^24,33–37^, we used concentrations of 0.5, 1, 2, and 3 mM METH in our studies. METH did cause an induction of cytoplasmic vacuoles (*n* = 12, *F* = 4.5, P < 0.05), which is an indication of cell injury, in the treated cells compared to the control (Fig. 1); however, METH did not trigger significant alteration in any cellular parameters, such as cell viability (Fig. 2A) or ROS production (Fig. 2B) or NO release (Fig. 2C) or cell cycle progression (Fig. 2D).

### Alteration in cell morphology with chronic METH treatment

**Figure 3:**
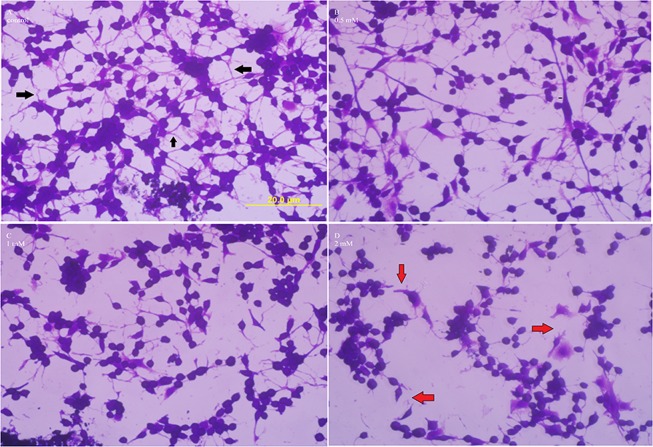
Effect of chronic METH on cell morphology. C6 astroglia-like cells were treated with equal volume of vehicle (PBS control, **A**) or 0.5 (**B**) or 1 (**C**) or 2 mM METH (**D**) for 48 h. Morphological images of crystal violet dye stained cells were taken using an inverted phase contrast IX-70 Olympus microscope with a 40x objective lens. Inter-cellular connections in control cells (**A**) are shown by black arrows, while their loss in 2 mM treated cells (**D**) is shown with red arrows. Scale bar: 20 μm.

Chronic treatment for 48 h was selected based on a previous study^38^. We initially focused on the chronic effect of METH on cell morphology because alteration in cell shape is considered an index of toxicity. After treating the cells with METH at 0.5, 1 and 2 mM for 48 h, the cells were stained with crystal violet^39^ and observed under the microscope. It was found that in the absence of external stimulants, the cells in 2.5% FBS in medium exhibited a high degree of differentiation. For instance, the control cells (Fig. 3A) exhibited neuronal morphology ranging from a polygonal to stellate shapes and displayed high branching of bi- or tri-polar neurite-like processes^40^ with extensive intercellular connections (Fig. 3A, black arrows). The morphology of cells treated with 0.5 mM METH remained the same as the control with extensive branching and intact intercellular joints (Fig. 3B); however, treatment with 1 and 2 mM METH gradually destroyed such associations (Fig. 3C,D, red arrows), causing most cells to become rounded. In addition, the cells treated with 2 mM METH lost the anchorage property, and the intercellular gaps expanded. Vacuoles were not observed in the treated cells at any concentration.

### Decreased cell viability

**Figure 4:**
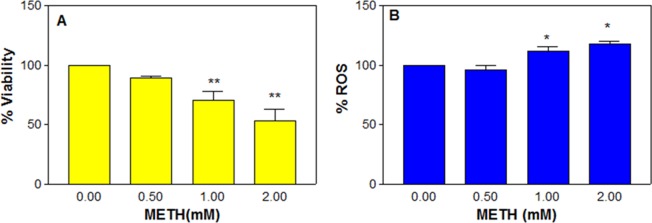
Effect of chronic METH in C6 astroglia-like cells on viability (**A**) and ROS generation (**B**). Cells were treated with an equal volume of vehicle (PBS control) or 0.5 to 2 mM METH for 48 h. Cell viability was evaluated by crystal violet dye (*n* = 6). ROS generation was measured by staining the cells with an H_2_DCFDA dye (20 μM, 30 min), followed by measurement of DCF in a micro plate fluorometer with excitation and emission filters set at 485 and 530 nm respectively (*n* = 11). Data were represented as mean ± SEM, *P < 0.05 or **P < 0.01, significant compared to corresponding controls, one-way ANOVA, Dunnett’s multiple comparison test.

METH concentrations below 500 μM did not show significant cell death even after 48 h treatment (data not shown); however, at higher concentrations like 0.5, 1 and 2 mM, there was a significant (*n* = 6, *F* = 10.8, P < 0.01) dose-dependent decrease in cell viability (Fig. 4A). The average cell viability was 89.5 ± 1.6, 70.2 ± 7.8 and 53.0 ± 9.7% of the control (100%) at 0.5, 1 and 2 mM, respectively.

### Increased ROS production

It was speculated that the dose-dependent decrease in cell viability (Fig. 4A) with METH treatment was related to ROS production. In order to confirm it, the cells were treated with METH at 0.5, 1, and 2 mM for 48 h and assessed for ROS release by dichlorodihydrofluorescein (DCF) measurement. It was found that there was a significant (*n* = 11, *F* = 17.9, P < 0.01) dose-dependent increase in ROS level compared to the control (Fig. 4B). The average increase in ROS at 1 and 2 mM METH was (±SEM) 111.9 ± 3.2 and 118.0 ± 1.9%, respectively compared to the control (100%).

### Increased lipid peroxidation

**Figure 5:**
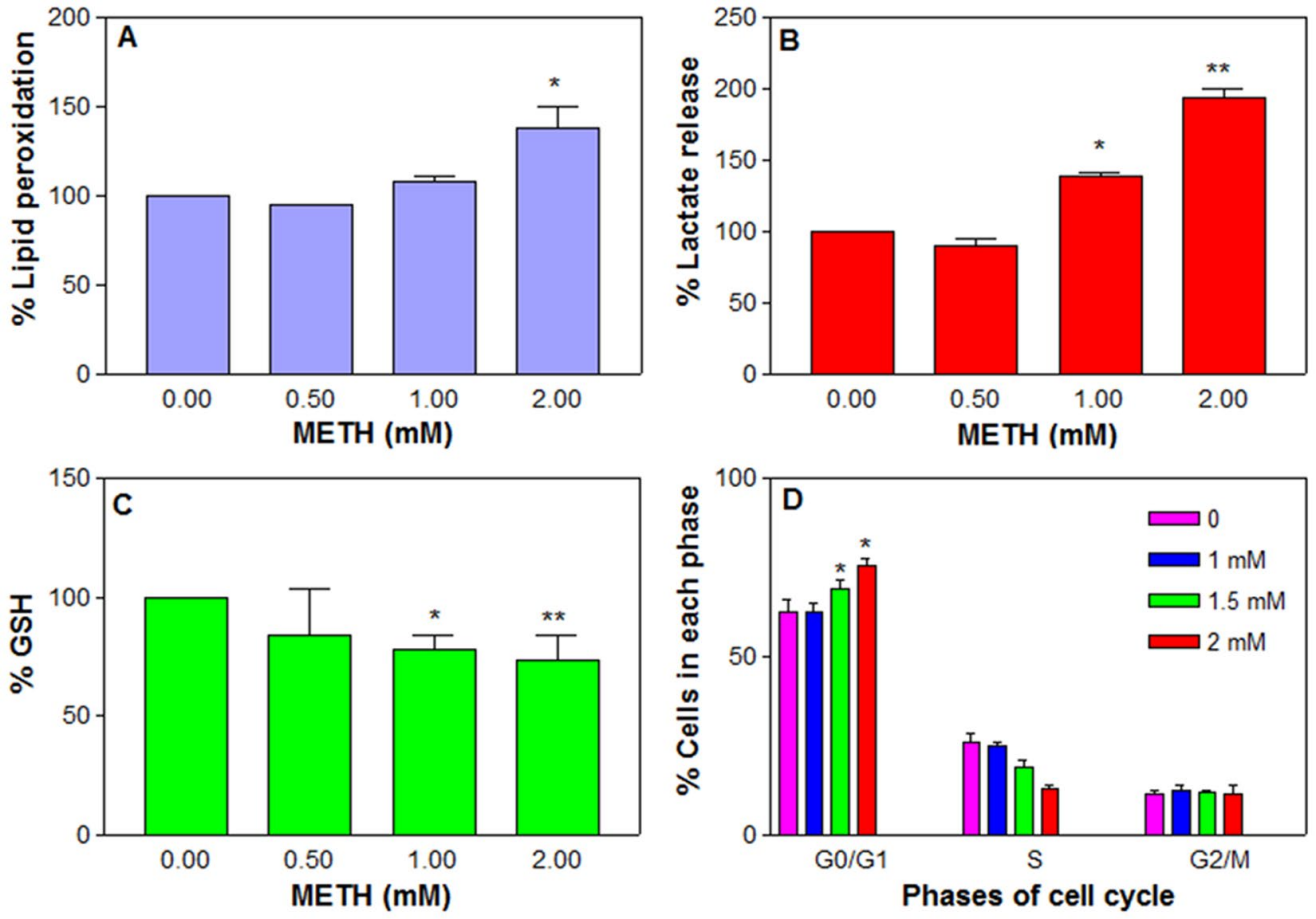
Effect of chronic METH on cells. C6 astroglia-like cells were treated with METH for 48 h. Lipid peroxidation (**A**, *n* = 6), lactate release (**B**, *n* = 9–12) and glutathione level (**C**, *n* = 6) were evaluated calorimetrically in a plate reader. For cell cycle analysis (**D**, *n* = 3), the cells were harvested and stained by PI staining solution for 1 h in the dark and analyzed by flow cytometry. Data were represented as mean ± SEM, *P < 0.05 or **P < 0.01, significant compared to control, one-way ANOVA, Dunnett’s multiple comparison test.

Since the dose-dependent increase in ROS can compromise the cell membrane integrity, we next measured the lipid peroxidation in the treated cells. There was a significant (*n* = 10, *F* = 13.1, P < 0.01) increase in the lipid peroxidation due to METH treatment compared to the control (Fig. 5A). The average peroxidation at 1 and 2 mM METH was ( ± SEM) 107.9 ± 2.6 and 137.4 ± 10.2%, respectively compared to the control.

### Increased lactate level

Treatment of cells with METH at 0.5, 1 and 2 mM for 48 h significantly (*n* = 12, *F* = 122.3, P < 0.01) and dose-dependently increased lactate release into the medium compared to the control (Fig. 5B). The average increase (±SEM) was 139.1 ± 2.8 and 193.7 ± 6.4% compared to the control (100%) at 1 and 2 mM METH, respectively.

### Decreased GSH level

Glutathione is one of the most available antioxidants in cells. In order to explore the potential role of METH on glutathione level, the cells were treated with METH at 0.5, 1 and 2 mM for 48 h. It was found that METH treatment caused a significant (*n* = 6, *F* = 18.5, P < 0.05) decrease in GSH level compared to the control (Fig. 5C). The GSH levels were (±SEM) 83.8 ± 19.5, 77.8 ± 5.5 and 73.6 ± 9.3% of the control (100%) at 0.5, 1 and 2 mM METH, respectively.

### METH inhibition at G0/G1 phase

It is widely known that proliferation is an extremely controlled event in the cells. In order to study the effect of METH on various phases of the cell cycle, cells were treated with METH at 1, 1.5 and 2 mM for 48 h and analyzed by flow cytometry. The data indicated that METH treatment caused a significant (*n* = 3, *F* = 4.9, P < 0.05) arrest in the G0/G1 phase (Fig. 5D). The percent cells accumulated at G0/G1 progression was significant at 1.5 mM (69.17 ± 2.2%) and 2 mM (75.4 ± 2.4%) METH compared to the control (62.4 ± 3.7%). This accumulation of cells correlated with a subsequent decrease in S phased cells at these concentrations. The cells at G2/M remained nearly the same at all METH treatments. METH-induced G0/G1 inhibition was reported earlier in dentate gyrus culture^41^.

### METH induces apoptosis

**Figure 6:**
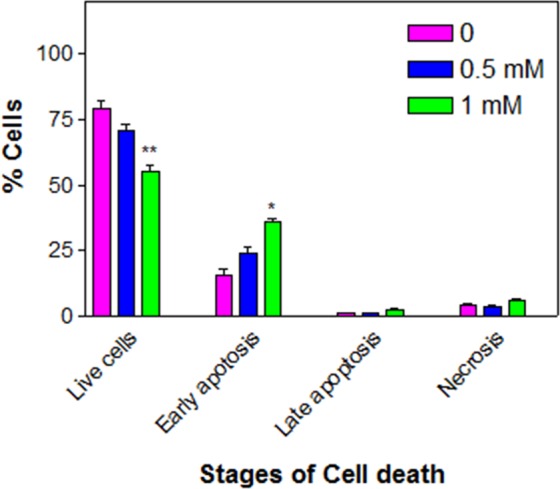
METH-induced apoptosis. C6 astroglia-like cells were treated with equal volume of vehicle (PBS control) or 0.5 or 1 mM METH for 48 h. For apoptosis assay (*n* = 3), the cells were harvested and stained by annexin-V-FITC and PI in the dark and analyzed by flow cytometry. Data were represented as mean ± SEM, *P < 0.05 or **P < 0.01, significant compared to control, one-way ANOVA, Dunnett’s multiple comparison test.

METH treatment gradually increased the early apoptotic cells in a significant (*n* = 3, *F* = 29.2, P < 0.05) and dose-dependent manner, which subsequently reflected in the gradual decrease in a number of live cells (Fig. 6). The average early apoptotic cells at 0.5 and 1 mM METH were (±SEM) 24.3 ± 2.1 and 35.9 ± 1.2%, respectively.

#### NAC pre-treatment prevents METH-induced cytotoxicity

**Figure 7:**
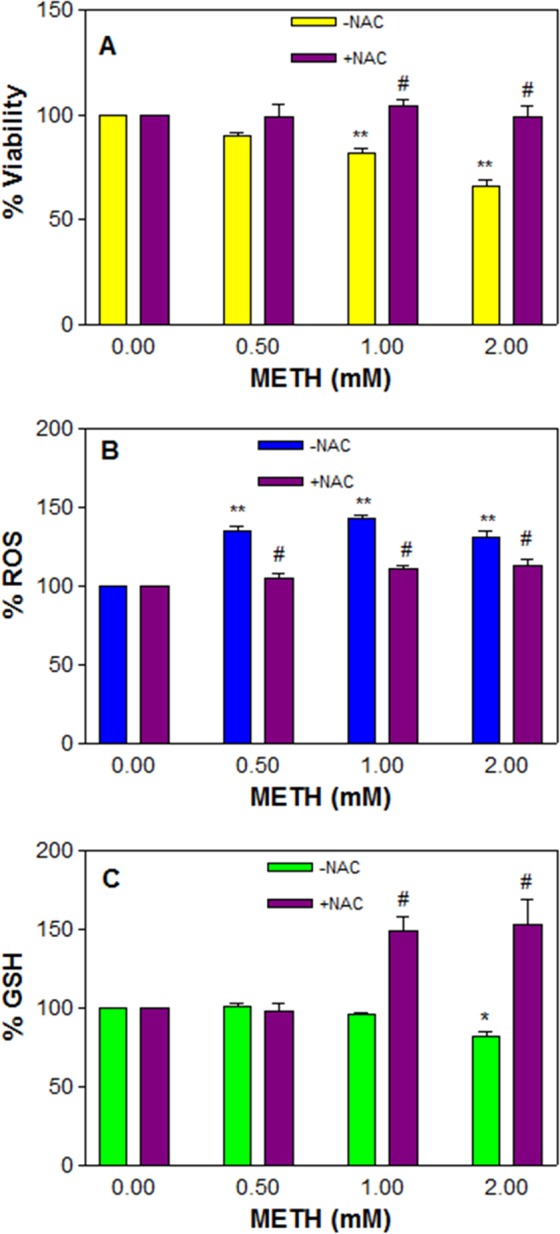
NAC rescues against METH-induced toxicity. C6 astroglia-like cells were pre-treated with 2.5 mM NAC or equal volume of vehicle (PBS control) for 1 h followed by co-treatment with 0.5, 1 and 2 mM METH for 48 h. Cell viability (*n* = 12) was evaluated by crystal violet dye (**A**), ROS generation (**B**, *n* = 7) by H_2_DCFDA (20 μM, 30 min) and glutathione level (**C**, *n* = 5) by Ellman’s reagent in a plate reader. Data were represented as mean ± SEM, *P < 0.05 or **P < 0.01, significant compared to control, or ^#^P < 0.01, significant compared to corresponding METH treatments, one-way ANOVA, Bonferroni’s multiple comparison test.

Rescue against METH-Induced cytotoxicity by NAC was evaluated for viability, ROS and GSH in the cells treated with METH for 48 h. Based on our previous studies^28^, we selected 2.5 mM NAC for 48 h exposure. Data indicated that NAC pre-treatment alone did not compromise cell viability, while it fully prevented the cells (*n* = 12, *F* = 17, P < 0.01) from METH-induced toxicity compared to METH alone treated cells (Fig. 7A). It was further observed that NAC pre-treatment decreased (*n* = 7, *F* = 35.9, P < 0.01) the METH-induced ROS generation (Fig. 7B) and increased (*n* = 5, *F* = 18.5, P < 0.01) the GSH level (Fig. 7C) compared to the corresponding METH alone treated cells. These data clearly indicate that NAC pre-treatment prevent METH-induced cytotoxicity.

## Discussion

Studies have demonstrated that substances of drug abuse cause psychological disorders by altering neuronal structure^42–44^ or mitochondrial function^45^ in the CNS. Given that these changes could last life-long^46^, they may have the propensity to manifest in several neurodegenerative diseases including Parkinson’s disease^47^, Schizophrenia, and Alzheimer disease. Since astrocytes insinuate between the neurons, interact in signal transmission, release trophic factors required for the growth^48^ and differentiation of neurons, METH-induced astrocytic death could potentially contribute to neuronal dysfunction and thereby lead to above neurodegenerative diseases. Several *in vivo*^49–52^ and *in vitro*^53^ studies showed that METH treatment induced astrogliosis, a sign of CNS injury, leading to modulation of neuronal activity^54^. Studies in human astrocytes and neurons further showed that METH toxicity was through glucose energy deprivation^55^. So far, there is no clear understanding of the mechanism of astrocyte death *in vivo* with METH exposure.

Direct assessment of METH toxic effect under *in vivo* is impeded due to body complexity. Employing primary cultures is not practical on account of restricted growth potential, finite life span and lack of cell homogeneity; thus, we employed C6 astroglia-like cells under *in vitro* conditions to gain insights on toxicity underlying cell death. These cells represent a good model system for astrocytes due to various merits outlined earlier^28–32^. These cells undergo differentiation and are shown to propagate calcium ion waves, called astrocyte excitability^56^, in the brain as well as under *in vitro* conditions^57,58^. Treatment with dibutyryl cAMP^59,60^ or taxol^54^ enabled these cells to differentiate, giving typical neuronal morphology. In our study, we found that C6 cells grown in reduced FBS (2.5%) without external growth factors induced a high level of differentiation, exhibiting neuronal morphology with extensive neurite-like processors and intercellular connections (Fig. 3A arrows). This observation is comparable with dibutyryl cAMP-induced differentiation in C6 cells^60^ but appears greater (Fig. 3A) than taxol-induced differentiation in the same cell line^61^.

The concentration of METH in plasma depends on several factors -like amount of drug intake, its frequency, drug tolerance, drug hydrolysis by blood esterases^62,63^, gender, genetics, age and time gap between drug intake & assessment. For example, METH level in serum after 3 h of intake was found to be 1.94 mg/L^64^, which is equal to 10.4 μM; (METH MW: 185.69), while the level was 6 μM after 22 h. It is important to know that these micro molar levels do not indicate consumption of METH in micro quantities by addicts. At the time of METH intake, its concentration in blood would be in milli molar range. For example, neurotoxic studies in rats were conducted^65^ at a maximum of 80 mg/kg METH as a binge dose (20 mg/kg, 4 times a day). In another study, these authors tested at 20 mg/kg/day METH for 10 days as a chronic dose in rats. Testing at 80 or 20 mg/kg in rats would translate to 8.11 or 2 mM METH respectively in the blood, taking a total volume of 12.8 ml/240 grams of rat weight (64 ml/kg). Similarly, in humans, a well adopted addict can use 1 g^66^ or more METH^62^ per day. On an average, 1 g translates to 1.07 mM METH in the blood assuming a total of 5 L. Since blood contains both plasma and corpuscles roughly in 1:1 ratio, the volume of plasma alone would now be equal to 2.5 L. Under this situation, METH concentration in plasma would be 2.14 mM. A comparison of highest METH concentration (2 mM) at chronic treatment in our study with a well tolerated human dose (1 g/day/2.5 L plasma = 2.14 mM) would reveal that the doses tested in our study (0.5, 1 and 2 mM) are within the range found in animals and humans.

Acute METH treatment did not induce cell death (Fig. 2A). Lack of ROS production or NO generation or cell cycle inhibition with acute METH treatment (Fig. 2B–D) corroborated with the lack of cell death (Fig. 2A). No studies were reported with acute METH effects for the induction of toxic markers in these cells except reports on activation of cells treated with METH^67^; similarly, the protective role of melatonin against METH-induced endoplasmic reticulum stress was reported^37^.

Lack of cell death (Fig. 2A) cannot be construed as METH’s inability to enter the cells, because METH treatment resulted in vacuolation (Fig. 1B), demonstrating its intracellular access. Vacuolation in our study is in agreement with earlier reports on different cell-types^68,69^. Despite vacuolation (Fig. 1B), lack of concomitant METH toxicity to cells (Fig. 2A) indicates that the formation of vacuoles in these cells was a secondary effect of toxicity. Although vacuolation is commonly associated with substances of drug abuse^28,39,70–73^, it was not noticed with chronic cocaine treatment in PC12 cells^38^ or with chronic METH treatment in C6 astroglia-like cells (Fig. 4B). On the other hand, vacuolation in mesencephalic cultures treated with chronic METH^68^ may suggest that this phenomenon is cell-type specific.

Chronic METH treatment in our study caused an alteration in cell morphology, destroyed the neurite-like processors (Fig. 3B, red arrows) and resulted in a decrease in viability (Fig. 4A), which corresponded with increased ROS production (Fig. 4B), an observation consistent with previous studies that showed METH-induced ROS generation in cultured astrocytes^74^. Furthermore, lactate release indicated that the treated cells were in the state of hypoxia (Fig. 5B), an alternate way for cell survival under mitochondrial dysfunction as shown earlier^75^. Increased ROS and dysfunctional mitochondria in our study are consistent with previous studies on long term METH exposure^76^. GSH is one of the most available antioxidants meant to cope with oxidative stress in cells. Consistent with the concept of the inverse relationship between increased ROS and decreased antioxidant levels in mammalian cells^77^, we found that the increased ROS level (Fig. 4B) with chronic METH treatment correlated with depletion of GSH level (Fig. 5C). This decrease was further paralleled with the decreased viability (Fig. 4A) and increased lipid peroxidation (Fig. 5A), suggesting that GSH depletion was one of the causes of cell death.

Increased ROS is associated with DNA damage^78,79^. Cells under such condition cease to proliferate in an attempt to repair DNA damage; however, if the damage is beyond repair, such cells undergo apoptotic death. Based on the observation of METH-induced cell death (Fig. 4A), increased ROS level (Fig. 4B), and inhibition of G0/G1 cell cycle phase (Fig. 5D), we suspected that the observed cell death could be apoptotic; further investigation proved that METH treatment indeed caused apoptosis in cells (Fig. 6), which is consistent with earlier reports^24,25,33^. Interestingly, NAC pre-treatment protected the cells completely (Fig. 7A) by decreasing the ROS (Fig. 7B) through increased GSH level (Fig. 7C), thus further supporting the results that increased ROS and consequent depletion of GSH were the main reasons of cell death in C6 cells treated with METH.

## Conclusion

Drug addicts often suffer from psychiatric illnesses such as depression^80^, anger, aggressiveness, and paranoia^81^. Damage to neuronal or astrocyte structures could be some of the contributing factors for these illnesses. In this study, we have shown that METH damages neurite-like processors (Fig. 3D). These data suggest that this damage could be a contributing factor to these illnesses.

It is disheartening to learn that METH is the main substance of drug abuse among pregnant women^82^. As per some estimations, its prevalence of use during pregnancy is ranged between 0.7–5.2%^83^, which is considered very high. Detrimental effects of METH on the developing fetus were reported earlier^84–88^. Thus, the impact of METH usage by pregnant women requires special national attention because there is a 3-fold increase in admissions of these women for METH treatment^82^, making this problem an economical burden to the society. The depletion in GSH^89^ due to increased oxidative stress^90^ is one of the causes of several psychiatric illnesses in METH addicts; therefore, compounds -such as NAC, which support GSH synthesis, could play a vital role in reducing the METH toxicity to neurons and alleviate from psychiatric-illnesses.

## Materials and Methods

### Materials

RPMI 1640, fetal bovine serum (FBS), penicillin/streptomycin sulfate, amphotericin B, phosphate-buffered saline (PBS) and L-glutamine were purchased from Media Tech (Herndon, VA). Crystal violet, L glutaraldehyde, trypan blue, methamphetamine (METH) hydrochloride (MW: 185.69), 2′,7′-dichlorofluorescin diacetate (H_2_DCFDA), 5,5′-dithiobis(2-nitrobenzoic acid) (DTNB), and EDTA were supplied by Sigma-Aldrich Company (St. Louis, MO). All other routine chemicals were of analytical grade.

### Cell culture maintenance

The CNS-derived rat C6 astroglia-like cell line (CCL-107) was purchased from American Type Culture Collection (Rockville, MD) and maintained as an adherent monolayer culture as described earlier^28^ in a humidified atmosphere containing 5% CO_2_ in air at 37 °C in an incubator. Cells were sub-cultured twice a week.

### METH and N-acetyl cysteine (NAC) treatments

The cells were seeded in 96-well microtiter plates at a starting density of 1 × 10^4^ cells per well in a total volume of 195 μl growth medium supplemented with 2.5% FBS. The cells were allowed to adhere to the wells in the incubator for 18–24 h before drug exposure. Stocks (1 M) and working stocks (20 to 120 mM) of METH were always prepared fresh in PBS. The cells, typically about 60–70% confluent, were treated with increasing concentrations of METH (0.5, 1, 2 and 3 mM for 1 h or 0.5, 1, and 2 mM for 48 h) in a final volume of 5 μl to prevent pH alteration of cell medium. Cells in medium alone or PBS in medium containing cells served as controls. Both controls and the treated samples were always present in the same culture plate. These plates were incubated for 1 or 48 h continuously without further renewal of growth media in a 5% CO_2_ at 37 °C incubator with plates capped in a normal fashion. For rescue experiments, the cells in 96-well plates were pretreated with 2.5 mM NAC for 1 h prior to METH co-treatment for 48 h. Cell viability, ROS and GSH levels were assayed at the end of the using methods outlined below.

### Microscopy

Morphology of crystal violet stained cells was evaluated as per the method described earlier^28^ using an inverted phase contrast IX70 Olympus microscope (Olympus, Ontario, NY) with a 40x objective. Photomicrographs were taken by a CCD camera (DP70, Olympus) with the image-acquisition system (DP-Controller, Olympus).

### Cell vacuolation

Cells were seeded in 96-well culture plates as described above. After treating with various concentrations of METH (0.5, 1, 2 and 3 mM) for 1 h, the cytoplasmic vacuoles in unstained cells were observed using an inverted phase contrast IX-70 Olympus microscope (Olympus) with a 40x objective. Photomicrographs were taken by an ocular video-camera system (MD35 Electronic eyepiece, Zhejiang Jincheng Scientific & Technology Co., Ltd., Hangzhou, China) using C-Imaging System Software (Compix Inc. Cranberry Township, PA). Vacuoles were quantified by neutral red dye uptake method^91^. This dye selectively deposits in the vacuoles besides leaving the cytoplasm unstained. The incorporated dye was extracted with 70% ethanol and 0.37% HCl, and the absorbance at 540 nm was taken in a micro plate reader.

### Viability assay

For acute treatment, the cell viability was evaluated using a Celltiter 96 Aqueous one solution kit (MTS, 10 μl, Promega, Madison, WI) as per the instructions provided by the manufacturer. After 30 min incubation at 37 °C, absorbance was taken in a micro plate reader at 490 nm. Because of false positive result due to interaction between NAC and MTS (unpublished observation), cell viability for chronic treatment was evaluated by crystal violet dye-uptake method as described previously^92^. Absorbance was taken in a micro plate reader at 540 nm.

### Measurement of intracellular ROS

Cells in 96-well plates in media devoid of phenol red with 2.5% FBS were treated with METH at 0.5, 1, 2 and 3 mM for 1 h or 0.5, 1 and 2 mM for 48 h followed by staining with the cell permeable dye H_2_DCFDA (20 μM final) for 30 min. After gentle washing and air drying of the cells, PBS (100 μl/well) was added. The fluorescent DCF was measured in an automatic fluorometer micro plate reader with the excitation filter set at 485 nm and the emission filter at 530 nm respectively (Synergy HTX multimode micro plate reader, BioTek Instruments, Winooski, VT).

### NO assay

Cells (2 × 10^4^ cells/well) were seeded in 96-well titer plates in media devoid of phenol red with 2.5% FBS. Next day, the cells were treated with METH at various concentrations (0.5, 1, 2 and 3 mM) for 1 h. At the end of incubation, 50 μl of media was transferred into a new plate and mixed with an equal volume of Griess reagent (1% sulfanilamide/0.1% N-naphthyl-ethylenediamine in 5% phosphoric acid) followed by a 10 min incubation in the dark. The absorbance at 546 nm was measured in a microtiter culture plate reader.

### Lipid peroxidation assay

Cells were seeded at a starting density of 0.5 × 10^6^ cells per well in 6-well culture plates in media devoid of phenol red with 2.5% FBS. After METH treatment at 0.5, 1 and 2 mM for 48 h, the cells were harvested and centrifuged at 13,000 rpm on a table top micro centrifuge for 6 min. All cell pellets were sonicated in PBS on ice for 3 sec and transferred into glass tubes. The lysates were further processed as per the earlier report^75^ and absorbance at 535 nm was measured in a micro plate reader. Clear medium without cells was used as a blank.

### Lactate assay

After treating the cells with METH at different concentrations (0.5, 1 and 2 mM) for 48 h in 96-well plates in media containing 2.5% FBS, lactate release was determined calorimetrically as per the study reported earlier^38^. Absorbance was measured at 490 nm in a microtiter culture plate reader.

### GSH level

After treating with METH at 0.5, 1 and 2 mM for 48 h in 96-well microtiter plates, the cells were fixed with 0.25% glutaraldehyde for 30 min, followed by gentle washing and air drying. Total cellular GSH was assayed with Ellman’s reagent (DTNB) as per earlier study^93^. The absorbance was measured at 412 nm in a micro plate reader.

### Cell cycle analysis

Treatments with METH at 1, 1.5, 2, 2.5 and 3 mM for 1 h or 1, 1.5 and 2 mM for 48 h were performed in 100 mm plastic culture dishes. The cells were harvested and fixed in 95% ice-cold ethanol for at least 24 h at 4 °C. The next day, the tubes were centrifuged at 1217 g for 7 min and re-suspended in 100 μl ethanol. Cells were stained as described elsewhere^94^. The proportion of cells in each stage of the cell cycle was determined within 2 h by using FACSCalibur flow cytometer (BD Biosciences, San Jose, CA). First the instrument was aligned with Calibrite beads (BD Biosciences). Then, prior to cell cycle analysis, the linearity of the fluorescence pulse detector was checked using chicken erythrocyte nuclei (BD Biosciences) on a linear scale with Doublet Discrimination Module at a flow rate of 12 μl/min as per the protocol supplied by the manufacturer. A total of 10,000 individual gated events were analyzed separately. CellQuest Software was used for the acquisition of the data, and the results were displayed as histograms (FL2-A vs counts). The percentage of cells in each phase was determined by using ModFit LT 3.0 (Verity Software House, Topsham, ME).

### Apoptosis assay

Annexin V-FITC Apoptosis Detection Kit (BD Biosciences Pharmingen, San Diego, CA) was utilized to measure the percent of apoptosis. In brief, after treatment with 0.5 and 1 mM METH in triplicate wells in 6-well plates for 48 h, the cells were harvested by trypsinization, washed in PBS and stained with annexin V/propidium iodide (PI) as per the protocol supplied by the manufacturer except for the steps of fixation and RNase reported earlier^95^. Samples were analyzed within 1 h by using FACSCalibur flow cytometer (BD Biosciences). CellQuest Pro software was used for the acquisition of the data at a flow rate of 12 μl/min, and the results were displayed as quadrant dot plots in the log mode; the X-axis indicates the fluorescence of annexin-V (green), while Y-axis indicates PI (red). Unstained cells (double negative), annexin-V-FITC single stained cells, and PI single stained cells were used for quadrant settings (compensation). In this study, untreated (control) and treated samples were stained with both dyes. A total of 10,000 individual gated events were analyzed separately for each sample. Quadrant statistics were used to quantify the cell population in the quadrants.

### Statistical analysis

The experimental results were presented as mean ± SEM. Since there is only one variable in each experiment, the data were analyzed for significance by one-way ANOVA and compared by Dunnett’s or Bonferroni’s multiple comparison tests using GraphPad Prism Software, version 3.00 (San Diego, CA). The test values of P < 0.05 and P < 0.01 were considered significant and highly significant, respectively.
